# The cognitive relevance of non-lesional damage to cortical networks in people with multiple sclerosis

**DOI:** 10.1007/s00415-024-12240-4

**Published:** 2024-03-05

**Authors:** Eva A. Krijnen, Tommy A. A. Broeders, Samantha Noteboom, Maureen van Dam, Albulena Bajrami, Piet M. Bouman, Frederik Barkhof, Bernard M. J. Uitdehaag, Eric C. Klawiter, Ismail Koubiyr, Menno M. Schoonheim

**Affiliations:** 1 MS Center Amsterdam, Anatomy and Neurosciences, Amsterdam Neuroscience, Amsterdam UMC Location VUmc, 1007 MB Amsterdam, The Netherlands; 2 Department of Neurology, Massachusetts General Hospital, Harvard Medical School, Boston, MA 02114 USA; 3 Division of Neurology, Emergency Department, “S. Chiara” Hospital, Azienda Provinciale per i Servizi Sanitari (APSS), 38122 Trento, Italy; 4 MS Center Amsterdam, Radiology and Nuclear Medicine, Amsterdam Neuroscience, Vrije Universiteit Amsterdam, Amsterdam UMC Location VUmc, 1081 BT Amsterdam, The Netherlands; 5 Queen Square Institute of Neurology and Centre for Medical Image Computing, University College London, London, WC1E 6BT UK; 6 MS Center Amsterdam, Neurology, Amsterdam Neuroscience, Amsterdam UMC Location VUmc, 1081 HV Amsterdam, The Netherlands; 7 De Boelelaan 1108, 1081 HZ Amsterdam, The Netherlands

**Keywords:** Multiple sclerosis, Cognition, Cortical lesions, Diffusion, Networks

## Abstract

**Background:**

Cognitive impairment, a common and debilitating symptom in people with multiple sclerosis (MS), is especially related to cortical damage. However, the impact of regional cortical damage remains poorly understood. Our aim was to evaluate structural (network) integrity in lesional and non-lesional cortex in people with MS, and its relationship with cognitive dysfunction.

**Methods:**

In this cross-sectional study, 176 people with MS and 48 healthy controls underwent MRI, including double inversion recovery and diffusion-weighted scans, and neuropsychological assessment. Cortical integrity was assessed based on fractional anisotropy (FA) and mean diffusivity (MD) within 212 regions split into lesional or non-lesional cortex, and grouped into seven cortical networks. Integrity was compared between people with MS and controls, and across cognitive groups: cognitively-impaired (CI; ≥ two domains at *Z* ≤ − 2 below controls), mildly CI (≥ two at − 2 < *Z* ≤ − 1.5), or cognitively-preserved (CP).

**Results:**

Cortical lesions were observed in 87.5% of people with MS, mainly in ventral attention network, followed by limbic and default mode networks. Compared to controls, in non-lesional cortex, MD was increased in people with MS, but mean FA did not differ. Within the same individual, MD and FA were increased in lesional compared to non-lesional cortex. CI-MS exhibited higher MD than CP-MS in non-lesional cortex of default mode, frontoparietal and sensorimotor networks, of which the default mode network could best explain cognitive performance.

**Conclusion:**

Diffusion differences in lesional cortex were more severe than in non-lesional cortex. However, while most people with MS had cortical lesions, diffusion differences in CI-MS were more prominent in non-lesional cortex than lesional cortex, especially within default mode, frontoparietal and sensorimotor networks.

**Supplementary Information:**

The online version contains supplementary material available at 10.1007/s00415-024-12240-4.

## Introduction

Cognitive impairment is recognized as a prevalent and debilitating symptom in people with multiple sclerosis (MS), occurring in up to 65%, predominantly in information processing speed, learning and memory domains [1, 2]. To predict impairment and develop effective therapies, it is of utmost importance to identify correlates of cognitive decline. White matter lesions have been considered the dominant hallmark of inflammatory disease activity in MS; though, grey matter (GM) involvement is more recently recognized to occur early on and throughout the disease course. Disability progression and worsening of cognitive functioning cannot be strongly predicted by a single MRI marker, as white matter lesion volume [3].

Therefore, in recent years, MRI measures related to cognition shifted from the white matter to GM, with a large role for structural abnormalities, as cortical lesions (CLs) [4]. CLs can already occur in early stages of MS [5]. They are present in up to 50% of cortical regions, especially in progressive MS, and are located predominantly purely intracortically (types II-IV) and within sulci of frontal and temporal lobes [6–8]. Characteristics of the extent of CLs, such as their total number, volume and spatial distribution, have been shown to (partly) explain cognitive functioning in people with MS [9, 10]. The exact and independent role of focal GM damage in the development of cognitive impairment, including its pathophysiological mechanism, remains unclear. A common thread here is that the severity of damage in and surrounding lesions appears challenging to quantify using MR sequences available in the clinical setting to date. Changes in microstructural integrity shows potential to quantify this damage in both lesional and non-lesional cortex in the MS brain, and better relate to disability and cognitive impairment [6, 11, 12]. Thus far, studies focused either on pathophysiological aspects of focal cortical microstructural integrity [6] or overall disease progression [12]. Recent work studying the independent contribution of microstructural integrity in lesional and non-lesional cortex to cognitive impairment as measures of severity of MS-related damage, identified the integrity of non-lesional cortex and to a lesser extent the normal-appearing white matter as predictors of cognitive impairment instead of CL measures [11].

Along with focal cortical damage, MRI measures related to cognition focussed on the role of functional networks [13]. Recent functional MRI studies have shown the importance of the accruing destabilization of certain networks in the development of cognitive decline, e.g., the ventral (VAN) and dorsal attention (DAN), frontoparietal (FPN) and default mode networks (DMN) [13, 14]. The structural substrates of destabilization of functional networks relevant for cognition, however, remain unclear in MS. Thence, the impact of focal cortical demyelination and diffuse normal-appearing microstructural changes on cognition within these networks could provide leads to structural substrates. Albeit the topographical distribution of CLs has been assessed [7], the role and clinical relevance of their presence and microstructural effects specifically within functional networks have not yet been evaluated.

Given the known importance of lesional and normal-appearing tissue changes in MS, our aim was to evaluate cortical structural (network) integrity as related to CLs in MS. Next, we aimed to assess which pattern of integrity changes in functionally-connected regions, i.e., cortical brain networks, show largest impact on cognitive impairment.

## Material and methods

### Participants

This cross-sectional study as secondary analysis of prospectively-acquired data was approved by the institutional ethics review board. All participants provided written informed consent. One-hundred seventy-six people with clinically diagnosed MS, recruited between 2008 and 2012 [15], and 48 healthy controls were included. Inclusion criteria for people with MS were a diagnosis of relapsing–remitting or progressive MS and the presence of all MRI sequences. All patients fulfilled the 2017 revised McDonald criteria [16], and were relapse free and without steroid treatment for more than two months, regardless of the use of any disease-modifying therapy. Exclusion criteria for people with MS and healthy controls were the presence of other neurologic or neuropsychiatric brain disease, and contraindication to MRI.

### Clinical assessments

To asses physical disability in people with MS, the Expanded Disability Status Scale [17] was conducted by an experienced physician blinded to the imaging results. All participants underwent an expanded Brief Repeatable Battery of Neuropsychological tests [15]. Details regarding the evaluation of neuropsychological tests can be found in the “Methods Supplement”. Briefly, based on *Z*-scores of seven predefined cognitive domains (attention, information processing speed, working memory, visuospatial memory, verbal memory, executive functioning—cognitive flexibility and verbal fluency, and—inhibition), people with MS were considered cognitively-impaired (CI) if they performed below − 2.0 SD on two or more cognitive domains and mildly CI if they performed below − 1.5 SD on two or more cognitive domains. Remaining people with MS not fulfilling any of these criteria were classified as cognitively-preserved (CP) [15, 18]. An average cognition *Z*-score was calculated based on the *Z*-scores of individual domains.

### Image acquisition

All participants underwent 3 T MRI (GE Signa HDxt), using an 8-channel phased-array head coil (partially collected by M.M.S.). The protocol including a 3D T1-weighted (T1) fast spoiled gradient-echo sequence for atlas segmentation [repetition/echo/inversion time 7.8/3.0/450 ms, 12° flip angle, 0.94 × 0.94 × 1.0 mm^3^ voxel size], a 3D fluid attenuated inversion recovery (FLAIR) sequence for white matter lesion segmentation (repetition/echo/inversion time 8000/125/2350 ms, 0.98 × 0.98 × 1.2 mm^3^ voxel size), a 3D Double Inversion Recovery (DIR) for GM lesion segmentation [19] (repetition/echo/inversion time 8000/125/498–2100 ms, 1.12 × 1.12 × 1.2 mm^3^ voxel size), and diffusion-weighted imaging for estimation of microstructural GM integrity (repetition/echo time 13,000/91 ms, 90° flip angle, 2.0 × 2.0 × 2.4 mm^3^ voxel size, 30 volumes with non-colinear diffusion gradients at 1000 s/mm^2^ and five at 0 s/mm^2^).

### Data processing

Data processing was performed by E.A.K., S.N. and T.A.A.B. Details regarding white and grey matter and CL segmentation and diffusion MRI processing are reported in the “Methods Supplement”. Briefly, FLAIR images were used to segment white matter lesions in people with MS by means of k-Nearest-Neighbours approach with tissue type priors. In order to segment our GM regions, cortical surface reconstruction was carried out by FreeSurfer 7.0 (https://surfer.nmr.mgh.harvard.edu/) on the lesion-filled 3D-T1 images. After surface reconstruction, the cortical GM was parcellated into 210 cortical regions (105 in each hemisphere) based on the Brainnetome atlas [20]. In native T1 space, fourteen deep GM regions (seven in each hemisphere) were segmented using FSL FIRST, of which both hippocampi were added to the cortical atlas. This yielded a 3D-T1 atlas for each participant consisting of 212 regions. All regions were grouped into seven distributed networks [sensorimotor (SMN), VAN, DAN, FPN, DMN, visual and limbic networks].

According to the consensus guidelines developed by the MAGNIMS group [21], CLs were scored and segmented on DIR images by P.M.B. (experienced neuroscientist), blinded to the patient characteristics, yielding a CL mask for each MS participant for lesion volumes and spatial distribution. By combining the CL mask and atlas, we were able to assess CL presence and volume within each atlas region.

Diffusion images were pre-processed by T.A.A.B. and M.M.S. with QSIPrep version 0.14.3. Diffusion tensor fitting was applied to the pre-processed diffusion images, yielding fractional anisotropy (FA) and mean diffusivity (MD) maps. In the exploration of the diffusion tensor imaging measures within the intricate cortical GM for their clinical relevance in cognition, we have chosen MD alongside FA. This selection was based on findings of previous literature studying diffusivity in the GM [6, 22] and the capability of especially MD to encompass a wider spectrum of microstructural alterations, rendering them both potential versatile metrics for evaluating tissue integrity in the cortex in the context of cognition.

Considering the resolution of conventional diffusion-weighted data and the physiological cortical thickness of the human brain, addressing partial volume effects is an essential step for extracting most accurate data possible. To minimize the potential confounding by partial volume averaging effects in our analyses, we weighted mean FA and MD values within each atlas region by partial volume fraction estimations that were derived from the cortical FreeSurfer output. As regional network analyses of cortical diffusion tensor imaging measurements are complicated by local cortical non-uniformity of diffusivity values, preventing straightforward interpretation of regional diffusivity and cytoarchitecture [23], we calculated regional FA and MD *Z*-scores per atlas region based on data of healthy controls.

### Statistical analysis

All statistical analyses were performed with the use of Python (v3.8.3) and IBM SPSS statistics 28.0 (SPSS Inc., Chicago, IL, USA). Normality was checked by Kolmogorov–Smirnov testing and histogram inspection. Skewed data, i.e., CL volume, count and fraction as well as white matter lesion volume, were log-transformed for subsequent analyses. Non-parametric Mann–Whitney U tests were performed if variables had not yet achieved a normal distribution. All participant characteristics were expressed as N (%) for categorical variables, Mean (SD) for continuous normally-distributed variables or Median [Range] for ordinal or not-normally distributed variables. Group comparisons were performed with the use of univariate and multivariate linear models and multinomial logistic regression models. As previous work has shown a more severe cognitive phenotype in individuals who are older, male and have lower levels of education [2], all analyses were adjusted for age, sex and level of education. Within-subject integrity differences were assessed by paired t-tests. A detailed statistical workflow is described in the “Methods Supplement”. Of all analyses, test statistics with 95% confidence interval are reported. All *P*-values were Bonferroni-corrected for multiple comparisons, displayed as *P*_*corr*_ in the main text. *P*-values < 0.05 were considered statistically significant.

## Results

### Demographics

Table 1 shows demographics of included participants, including cognitive subgroups. Mean age of people with MS was 54 ± 9 compared to 51 ± 7 in healthy controls (P = 0.007). Of people with MS, 44 people with MS were defined as CI, 37 as mildly CI and 95 as CP. Mildly CI-MS and CI-MS were most severely impaired on attentional functioning [*Z* = − 1.52 (0.80), and *Z* = − 1.99 (1.48), respectively] and information processing speed [*Z* = − 2.03 (0.96), and *Z* = − 2.81 (1.29), respectively]. CI-MS were more severely physically disabled and had a higher white matter lesion load compared to CP-MS.

**Table 1:** Demographics of people with multiple sclerosis and healthy volunteers

	HC N = 48	MS N = 176	HC vs. MS^a^	CP N = 95	Mildly CI N = 37	CI N = 44	CP vs. mildly CI vs. CI^b^
Demographics							
Age (years)	50.83 (7.04)	54.24 (9.11)	t(94.4) = − 2.78, P = 0.007	53.45 (8.98)	54.73 (8.87)	55.53 (9.63)	F(2,173) = 0.85, P = 0.43
Sex (female), N (%)	27 (56.3)	118 (67.0)	Χ^2^(1) = 1.93, P = 0.17	64 (67.4)	25 (67.6)	29 (65.9)	Χ^2^(2) = 0.04, P = 0.98
High education^c^ (yes), N (%)	26 (54.2)	73 (41.5)	Χ^2^(1) = 2.46, P = 0.12	42 (44.2)	16 (43.2)	15 (34.1)	Χ^2^(2) = 1.33, P = 0.52
MS characteristics							
MS subtype (RR/SP/PP), N (%)		111(63.1)/42 (23.9)/23 (13.1)		65 (68.4)/19 (20.0)/11 (11.6)	21 (56.8)/11 (29.7)/5 (13.5)	25 (56.8)/12 (27.3)/7 (15.9)	Χ^2^(4) = 2.72, P = 0.61
Disease duration (months)		200.6 (68.1)		198.8 (66.2)	213.1 (77.3)	193.7 (64.1)	F(2,165) = 0.84, P = 0.43
EDSS		4 [1.5–8]		3 [1.5–8]	4 [2–8]	4.3 [1.5–7.5]	H(2) = 14.00, P = 0.001
Medication, N (%)		52 (29.5)		25 (26.3)	16 (43.2)	11 (25.0)	Χ^2^(2) = 4.25, P = 0.12
First-line		39 (22.2)		20 (21.2)	11 (29.7)	8 (18.2)	
Second-line		13 (7.4)		5 (5.3)	5 (38.5)	3 (23.1)	
Neuropsychological functioning, *Z*-score^d^							
Average cognition	0.00 (0.43)	– 0.74 (0.87)	t(156.2) = 8.20, P < 0.001	– 0.19 (0.49)	– 0.94 (0.32)	– 1.75 (0.82)	F(2,173) = 116.74, P < 0.001
Attention	0.00 (0.60)	– 1.03 (1.17)	t(151.4) = 8.26, P < 0.001	– 0.41 (0.63)	– 1.52 (0.80)	– 1.99 (1.48)	F(2,168) = 46.96, P < 0.001
Information processing speed	0.00 (1.00)	– 1.49 (1.44)	t(106.4) = 8.26, P < 0.001	– 0.67 (1.06)	– 2.03 (0.96)	– 2.81 (1.29)	F(2,171) = 61.92, P < 0.001
Verbal memory	0.00 (0.89)	– 0.51 (1.11)	t(220) = 2.96, P = 0.003	0.06 (0.93)	– 0.98 (0.79)	– 1.39 (0.96)	F(2,171) = 43.82, P < 0.001
Visuospatial functioning	0.00 (0.95)	– 0.62 (1.15)	t(222) = 3.45, P < 0.001	– 0.24 (1.04)	– 0.64 (0.91)	– 1.45 (1.15)	F(2,173) = 20.44, P < 0.001
Working memory	0.00 (1.00)	– 0.36 (1.30)	t(212) = 1.76, p = 0.08	– 0.06 (0.92)	– 0.36 (1.40)	– 1.03 (1.67)	F(2,164) = 8.48, P < 0.001
EF–inhibition	0.00 (1.00)	– 0.49 (1.43)	t(214) = 2.21, P = 0.03	0.04 (1.25)	– 0.61 (1.10)	– 1.58 (1.45)	F(2,165) = 22.69, P < 0.001
EF–CF & verbal fluency	0.00 (0.77)	– 0.54 (1.21)	t(116.0) = 3.73, P < 0.001	– 0.05 (0.75)	– 0.34 (0.67)	– 1.76 (1.49)	F(2,173) = 47.42, P < 0.001
MRI characteristics							
NB volume	0.73 (0.02)	0.69 (0.04)	t(143.5) = 7.15, P < 0.001	0.71 (0.04)	0.69 (0.04)	0.67 (0.04)	F(2,173) = 13.94, P < 0.001
NCGM volume	0.39 (0.02)	0.38 (0.02)	t(222) = 2.79, P = 0.006	0.39 (0.02)	0.38 (0.02)	0.37 (0.02)	F(2,173) = 7.04, P = 0.001
NDGM volume	3.64 × 10^–2^ (1.84 × 10^–3^)	3.29 × 10^–2^ (3.56 × 10^–3^)	t(149.6) = 9.07, P < 0.001	3.42 × 10^–2^ (3.33 × 10^–3^)	3.22 × 10^–2^ (3.06 × 10^–3^)	3.08 × 10^–2^ (3.35 × 10^–3^)	F(2,173) = 16.48, P < 0.001
WM lesion volume (mL)		17.77 (14.55)		12.99 (10.04)	22.23 (17.65)	24.34 (16.39)	F(2,173) = 10.97, P < 0.001^e^

Demographics of healthy volunteers (HC) and people with multiple sclerosis (MS), including cognitive subgroups: cognitively preserved (CP), mildly cognitively impaired (CI) and CI people with multiple sclerosis (MS). Variables are reported as Mean (SD) or Median [Range] unless otherwise indicated. *RR* Relapsing remitting; *SP* secondary progressive; *PP* primary progressive; *EDSS* expanded disability status scale; *EF* executive functioning; *CF* cognitive flexibility; *NB* normalized brain; *NCGM* normalized cortical grey matter volume; *NDGM* normalized deep grey matter volume; *WM* white matter

^a^Independent t-test (continuous variables) or Chi-square test (categorical variables). Test statistics with corresponding* P*-values are reported

^b^Univariate linear model (continuous variables) or Chi-square test (categorical variables). Test statistics with corresponding* P*-values are reported

^c^High level of education was defined as educational level corresponding to ≥ 6 on the Dutch Verhage scale

^d^Neuropsychological assessment data was missing in 5.1% (*N* = 9) of people with MS for the domain working memory, in 4.5% (*N* = 8) for EF–inhibition, in 2.8% (*N* = 5) for attention, in 1.1% (*N* = 2) for information processing speed and for verbal memory; no missing data for visuospatial memory and EF–CF & verbal fluency

^e^Comparison between cognitive subgroups performed on log-scale

### Cortical lesion distribution

Table 2 shows the volumes and presence of CL in people with MS. CLs were present in 87.5% of people with MS. CL count and volumes were lower in CP-MS compared to mildly CI-MS [40.2% increase in CL count in mildly CI-MS; OR (95% confidence interval) = 3.55 (1.56; 8.08), *P*_*corr*_ = 0.005, and 27.8% increase in CL volume in mildly CI-MS; OR (95% confidence interval) = 1.91 (1.18; 3.10), *P*_*corr*_ = 0.02, respectively] and CI-MS [48.8% increase in CL count in CI-MS; OR (95% confidence interval) = 5.09 (2.23; 11.60), *P*_*corr*_ < 0.001, and 26.9% increase in CL volume in CI-MS; OR (95% confidence interval) = 1.88 (1.20; 2.95), *P*_*corr*_ = 0.01, respectively]. The CL load relative to network volume was highest in the VAN [1.03 × 10^–3^% (0.00; 26.84 × 10^–3^%)], followed by the limbic network [0.84 × 10^–3^% (0.00; 11.52 × 10^–3^%)] and DMN [0.46 × 10^–3^% (0.00; 11.38 × 10^–3^%)]. At a network level, the percentage of people with MS with at least one CL was greater in mildly CI-MS compared to CP-MS in the SMN [57.9% of CP-MS and 86.5% of mildly CI-MS; OR (95% confidence interval) = 4.84 (1.71; 13.65), *P*_*corr*_ = 0.04], and in CI-MS compared to CP-MS in the visual network [23.2% of CP-MS and 39.7% in CI-MS; OR (95% confidence interval) = 4.55 (2.08; 9.95), *P*_*corr*_ = 0.002]. Compared to CP-MS, the relative CL load was higher in CI-MS in all, except the VAN (*P*_*corr*_–range =  < 0.001–0.02), and higher in mildly CI-MS in SMN and limbic network (*P*_*corr*_ = 0.02, and *P*_*corr*_ = 0.009, respectively; Table 2).

**Table 2:** Distribution of regions with cortical lesions

	MS N = 176	CP N = 95	Mildly CI N = 37	CI N = 44	CP vs mildly CI^a^	CP vs CI^a^
Global CL characteristics						
Presence of ≥ 1 CL	154 (87.5%)	80 (84.2%)	35 (94.6%)	39 (88.6%)	3.24 (0.70–15.06), P = 0.13	1.44 (0.48–4.31), P = 0.52
CL count	9 [0–123]	6 [0–74]	13 [0–68]	15.5 [0–123]	3.55 (1.56–8.08), P = 0.003*	5.09 (2.23–11.60), P < 0.001*
Total CL volume, mL	0. 27 [0.00–6.76]	0.17 [0.00–4.62]	0.26 [0.00–3.68]	0.69 [0.00–6.76]	1.91 (1.18–3.10), P = 0.009*	1.88 (1.20–2.95), P = 0.006*
Functional networks						
VAN						
Presence of ≥ 1 CL	123 (69.9%)	61 (49.6%)	27 (73.0%)	35 (79.5%)	1.47 (0.63–3.45), P = 0.37	2.17 (0.92–5.12), P = 0.08
Fraction of CL, × 10^–3^%	1.03 [0.00–26.84]	0.61 [0.00–2.26]	1.21 [0.00–26.84]	1.35 [0.00–21.06]	U = 1437.0^b^, P = 0.10	U = 1575.0^b^, P = 0.02
DAN						
Presence of ≥ 1 CL	107 (60.8%)	48 (50.5%)	26 (70.3%)	33 (75.0%)	2.48 (1.08–5.72), P = 0.03	3.36 (1.48–7.66), P = 0.004
Fraction of CL, × 10^–3^%	0.39 [0.00–29.15]	0.04 [0.00–19.16]	0.81 [0.00–14.32]	0.94 [0.00–29.15]	U = 1269.5^b^, P = 0.01	U = 1416.5^b^, P = 0.002*
SMN						
Presence of ≥ 1 CL	122 (69.3%)	55 (57.9%)	32 (86.5%)	35 (79.5%)	4.84 (1.71–13.65), P = 0.003*	2.95 (1.25–6.95), P = 0.01
Fraction of CL, × 10^–3^%	0.35 [0.00–18.50]	0.16 [0.00–13.23]	0.61 [0.00–13.10]	1.43 [0.00–18.50]	U = 1146.0^b^, P = 0.002*	U = 1275.0^b^, P < 0.001*
Visual network						
Presence of ≥ 1 CL	63 (35.8%)	22 (23.2%)	16 (43.2%)	25 (39.7%)	2.64 (1.16–5.99), P = 0.02	4.55 (2.08–9.95), P < 0.001*
Fraction of CL, × 10^–3^%	0.00 [0.00–9.86]	0.00 [0.00–6.26]	0.00 [0.00–6.79]	0.06 [0.00–9.86]	U = 1339.5^b^, P = 0.008	U = 1330.5^b^, P < 0.001*
Limbic network						
Presence of ≥ 1 CL	117 (66.5%)	53 (55.8%)	29 (78.4%)	35 (79.5%)	2.92 (1.20–7.11), P = 0.02	3.22 (1.37–7.57), P = 0.007
Fraction of CL, × 10^–3^%	0.84 [0.00–11.52]	0.34 [0.00–10.34]	1.54 [0.00–11.52]	1.50 [0.47–10.50]	U = 1102.0^b^, P < 0.001*	U = 1353.0^b^, P < 0.001*
DMN						
Presence of ≥ 1 CL	133 (75.6%)	64 (67.4%)	31 (83.8%)	38 (86.4%)	2.64 (0.98–7.11), P = 0.06	3.35 (1.25–9.00), P = 0.02
Fraction of CL, × 10^–3^%	0.46 [0.00–11.38]	0.28 [0.00–10.82]	0.77 [0.00–8.66]	1.25 [0.00–11.38]	U = 1200.0^b^, P = 0.004	U = 1231.0^b^, P < 0.001*
FPN						
Presence of ≥ 1 CL	104 (59.1%)	46 (48.4%)	26 (70.3%)	32 (72.7%)	2.64 (1.16–6.03), P = 0.02	3.15 (1.42–7.01), P = 0.005
Fraction of CL, × 10^–3^%	0.23 [0.00–22.51]	0.00 [0.00–12.93]	0.42 [0.00–4.77]	0.83 [0.00–22.51]	U = 1307.5^b^, P = 0.02	U = 1292.0^b^, P < 0.001*

Distribution of cortical lesions (CL) across functionally related regions in people with multiple sclerosis (MS), divided in three cognitive subgroups: cognitively preserved (CP), mildly cognitively impaired (CI) and CI patients. Fraction of CL is based on the volume of CLs relative to total network volume. The variables reflecting the count, volume and fraction of CL were log(x + 1)-transformed before group comparisons. Presence of CL are shown as N (%). Count, fraction and volume of CL are shown as Median [Range]. Raw unadjusted* P*-values are shown

*VAN* Ventral attention network; *DAN* dorsal attention network; *SMN* sensorimotor network; *DMN* default mode network; *FPN* frontoparietal network

*P-value surviving Bonferroni correction (*P* < 0.025 for global CL characteristics, and *P* < 3.57 × 10^–3^ for within-network comparisons)

^a^Multinomial logical regression, adjusting for age, sex and level of education. CP is used as reference category. Odds ratio’s with 95% confidence interval are reported, with corresponding* P*-value

^b^Mann-Whitney Test due to not-normally distributed log(x + 1)-transformed variable

Of 212 cortical regions, a median of 13 (6.1%; range 0–134) regions were defined as region with lesional cortex (Supplementary Fig. 1).

### People with MS versus healthy controls: non-lesional cortical FA and MD

Mean MD in non-lesional cortex was significantly increased in people with MS compared to healthy controls [*F*(1,219) = 19.88, *η*^*2*^ = 0.08, *P*_*corr*_ < 0.001; Table 3]. Mean FA in non-lesional cortex did not differ between people with MS and healthy controls [*F*(1,219) = 2.81, *η*^*2*^ = 0.01, *P*_*corr*_ = 0.19].

**Table 3:** Integrity measures in normal-appearing and lesions cortex

Integrity measure	HC N = 48	MS N = 176	HC vs. MS^a^
Mean diffusivity			
NA cortex	1.02 (0.04)	1.06 (0.05)	F(1,219) = 19.88, P < 0.001*
Lesional cortex	–	1.08 (0.08)	
Fractional anisotropy			
NA cortex	0.18 (0.01)	0.17 (0.01)	F(1,219) = 2.81, P = 0.10
Lesional cortex	–	0.18 (0.01)	

Integrity measure	CP N = 95	Mildly CI N = 37	CI N = 44	Group difference^b^	Post-hoc CP vs. CI^c^
Mean diffusivity					
Overall	1.05 (0.05)	1.07 (0.04)	1.08 (0.06)	F(2,170) = 5.70^d^, P = 0.004*	
NA cortex	1.05 (0.04)	1.07 (0.04)	1.09 (0.05)		F(1,114) = 11.52, P < 0.001*
Lesional cortex	1.07 (0.08)	1.08 (0.06)	1.11 (0.09)		F(1,114) = 6.84, P = 0.01*
Fractional anisotropy					
Overall	0.17 (0.01)	0.18 (0.01)	0.17 (0.01)	F(2,170) = 0.51, P = 0.60	
NA cortex	0.17 (0.01)	0.17 (0.01)	0.17 (0.01)		
Lesional cortex	0.18 (0.02)	0.18 (0.01)	0.18 (0.01)		

Raw integrity values in normal-appearing (NA) and lesional cortex of people with multiple sclerosis (MS) and in NA cortex in healthy controls (HC; upper panel), and per cognitive subgroup (lower panel): cognitively preserved (CP), mildly cognitively impaired (CI) and CI patients. Variables are reported as Mean (SD). Raw unadjusted P-values are shown

As people with MS without CLs were excluded from regional subanalyses (as noted in^b^), the means reported in the main table are based on a different sample of the subgroups than reported in the table: CP-MS *N* = 80, Mildly CI-MS *N* = 35, and CI-MS *N* = 39

*P-value surviving Bonferroni correction (*P* < 0.025)

^a^Multivariate linear model, adjusting for age, sex and high level of education. F-test statistics with corresponding* P*-values are reported

^b^Univariate linear model for whole-brain integrity, adjusting for age, sex and high level of education. F-test statistics with corresponding* P*-values are reported

^c^Multivariate linear model for whole-brain integrity in both NA cortex and lesional cortex, adjusting for age, sex and high level of education, as post-hoc analysis for CP-MS versus CI-MS. Using this model, only people with MS with CLs were included, eliminating potential bias from people with MS without CLs. F-test statistics with corresponding* P*-values are reported

^d^Bonferroni-corrected significant difference between CP-MS and CI-MS

### Within-subject integrity differences: lesional versus non-lesional cortex

**Fig. 1:**
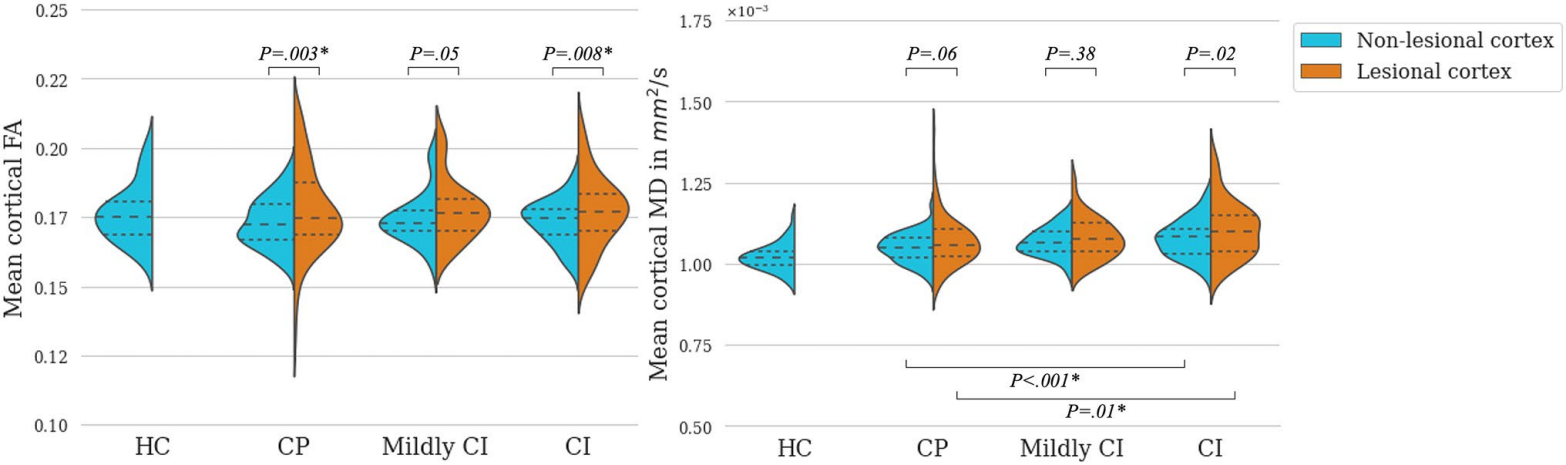
Cortical microstructural integrity measures in included participants. Mean diffusivity (MD) and fractional anisotropy (FA) in regions with non-lesional cortex in healthy controls (HC) and non-lesional and lesional cortex in people with multiple sclerosis (MS), divided into three cognitive subgroups: cognitively impaired (CI), mildly CI, cognitively preserved (CP) people with multiple sclerosis (MS). Inner lines denote quartiles (25–50–75%). Raw unadjusted *p*-values are shown. *P*-values surviving Bonferroni correction are marked with an asterisk (*)

In MS, within-subject mean FA and MD were significantly increased in regions with CL compared to those containing only non-lesional cortex [*t*(153) = 4.44, *P*_*corr*_ = 3.40 × 10^–5^ and* t*(153) = 2.95, *P*_*corr*_ = 0.007, respectively]. Subanalysis in cognitive subgroups showed significantly increased FA in regions with CL compared to non-lesional cortex in CP-MS and CI-MS [t(79) = 3.10, *P*_*corr*_ = 0.016 and t(38) = 2.79, *P*_*corr*_ = 0.049, respectively; Fig. 1 and Supplementary Table 2]. Mean MD was not significantly increased in regions with CL compared to non-lesional cortex within cognitive subgroups (Fig. 1 and Supplementary Table 2).

### Cognitive subgroup comparisons: lesions and non-lesional cortex

Mean cortical MD varied significantly between cognitive subgroups [*F*(2,170) = 5.70, *η*^*2*^ = 0.06, *P*_*corr*_ = 0.008; Table 3]. Post-hoc analyses showed a significant increase in cortical MD in CI-MS compared to CP-MS [Mean difference (95% confidence interval) = 0.029 (0.007; 0.051), *P*_*corr*_ = 0.009]. Mildly CI-MS showed no effect compared to either CP or CI-MS. Regional subanalyses showed a significant increase in mean MD of non-lesional cortex as well as in regions with CL in CI-MS compared to CP-MS [*F*(1,114) = 11.52, *η*^*2*^ = 0.09, *P*_*corr*_ = 0.002 and *F*(1,114) = 6.84, *η*^*2*^ = 0.06, *P*_*corr*_ = 0.02, respectively; Fig. 1]. Mean cortical FA did not differ between cognitive subgroups [*F*(2,170) = 0.51, *η*^*2*^ = 0.01, *P* = 0.60].

As we only detected integrity alterations in MD between CP-MS and CI-MS, subsequent analyses focused on alterations in MD between these two cognitive subgroups. Also, in order to avoid bias from physiological integrity variances throughout the cortex, the following spatial network analyses were performed with the use of the *Z*-scores of mean MD instead of the raw mean values used in the global analyses above.

### MD in networks: CI vs CP

#### Group comparisons

**Fig. 2:**
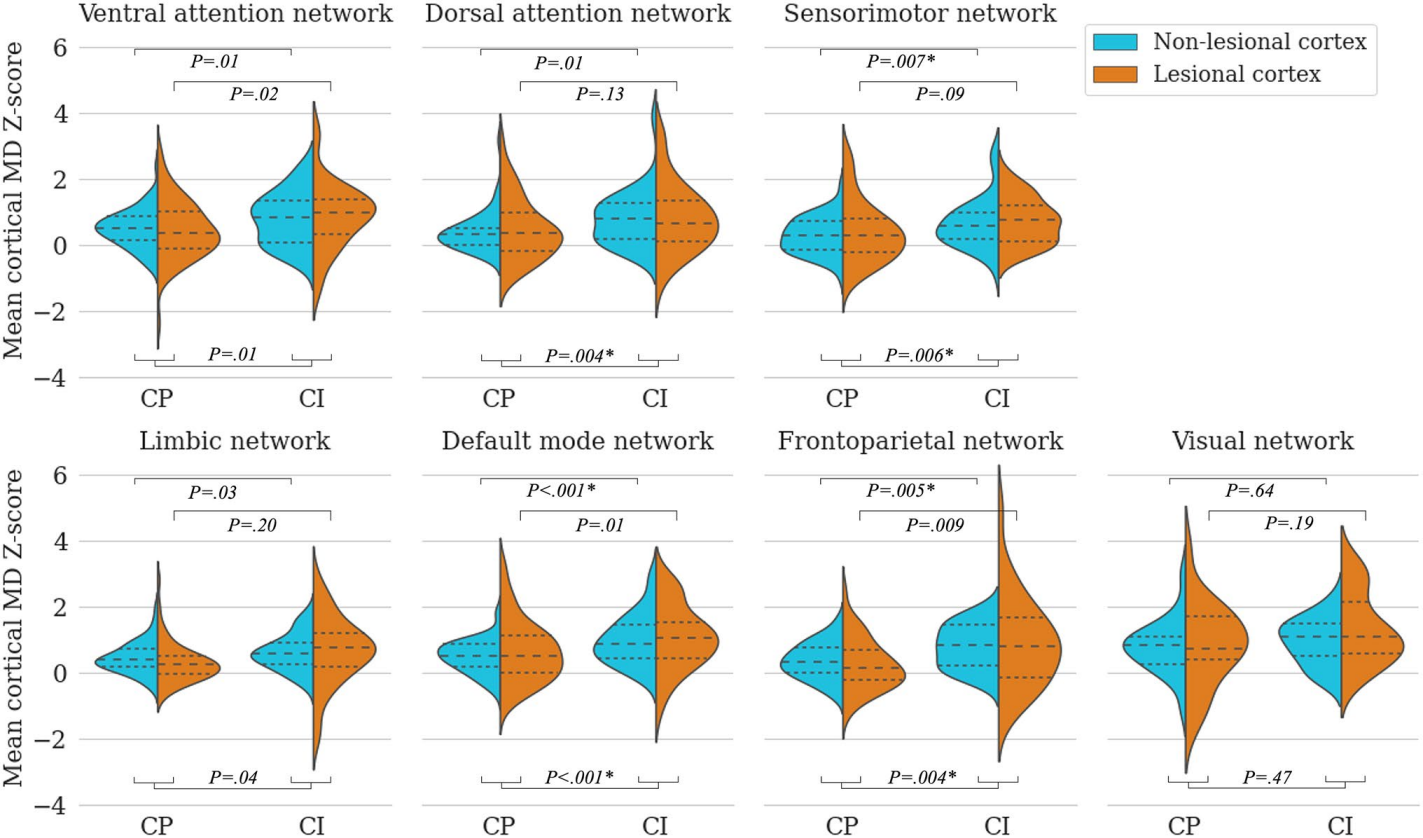
Cortical microstructural integrity measures across functional networks in cognitive groups. Mean diffusivity (MD) *Z*-scores in regions with non-lesional and lesional cortex in cognitively preserved (CP) and cognitively impaired (CI) people with multiple sclerosis (MS) across functional networks. As no integrity alterations in cortical MD were detected in mildly CI-MS compared to either CP-MS or CI-MS, subsequent network analyses shown here focused on alterations in MD between CP-MS and CI-MS. Inner lines denote quartiles (25–50–75%). Raw unadjusted *p*-values are shown. *P*-values surviving Bonferroni correction are marked with an asterisk (*)

**Fig. 3:**
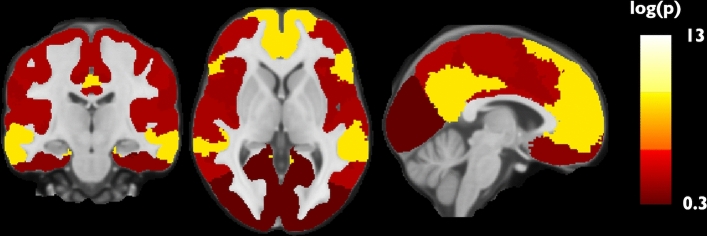
Visualization of the P-value distribution for the regional network differences in mean diffusivity *Z*-scores in people with multiple sclerosis versus healthy control. P-values were log-transformed to normalize the distribution and increase distinctiveness between networks

All networks, except the visual network, showed increases in MD *Z*-score in CI-MS compared to CP-MS, of which increases in the DAN [*F*(1,76) = 8.89, *η*^*2*^ = 0.11, *P*_*corr*_ = 0.03], SMN [*F*(1,85) = 8.02, *η*^*2*^ = 0.09, *P*_*corr*_ = 0.04], DMN [*F*(1,97) = 14.12, *η*^*2*^ = 0.13, *P*_*corr*_ = 0.002] and FPN [*F*(1,73) = 8.74, *η*^*2*^ = 0.11, *P*_*corr*_ = 0.03] survived Bonferroni correction (Figs. 2 and 3). Looking at non-lesional cortex separately, CI-related MD increases compared to CP-MS were seen in FPN, SMN and DMN [*F*(1,73) = 8.46, *η*^*2*^ = 0.10, *P*_*corr*_ = 0.03, *F*(1,85) = 7.66, *η*^*2*^ = 0.08, *P*_*corr*_ = 0.048, and *F*(1,97) = 15.55, *η*^*2*^ = 0.14, *P*_*corr*_ = 0.001, respectively; Fig. 2]. *Z*-score increases in mean MD in regions with CL did not survive Bonferroni correction (Supplementary Table 3).

#### Regression analysis

**Fig. 4:**
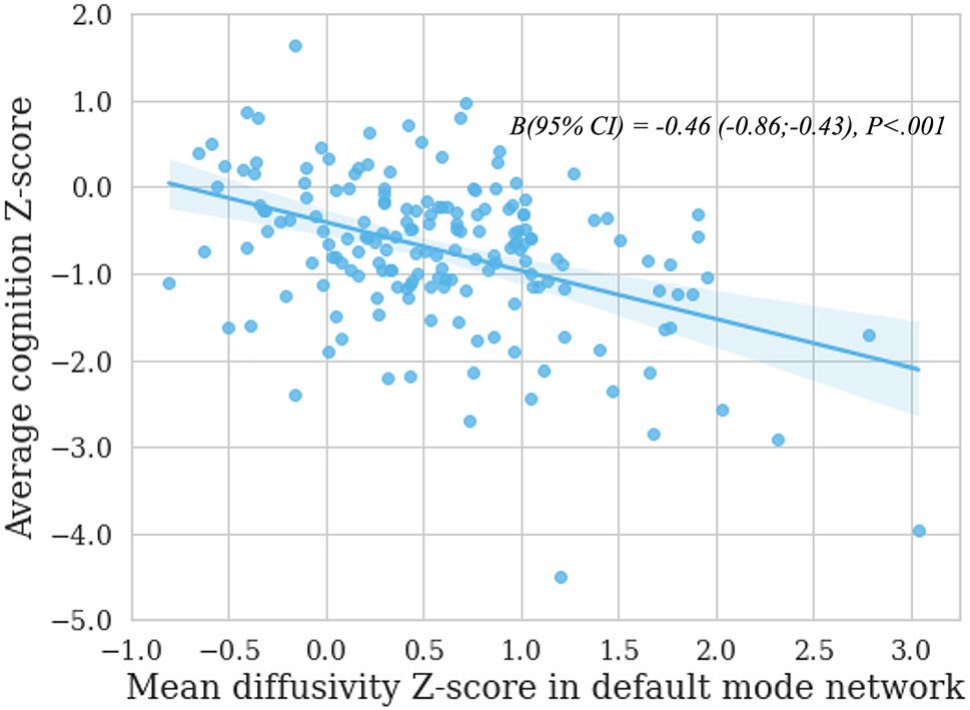
Association between mean diffusivity in the default mode network and average cognition in people with multiple sclerosis. Integrity (x-axis) and cognition (y-axis) measures shown in the figure are transformed to *Z*-scores based on data of the included healthy controls. Standardized Beta-coefficients (β) with 95% confidence interval (CI) are reported with corresponding *p*-value

The non-lesional cortex within networks showing significant CI-related MD increases were selected as candidate markers to explain average cognition, in order to study in which regions integrity differences could best reflect cognitive functioning in MS. Therefore, the MD *Z*-scores of the non-lesional cortex of the FPN, SMN and DMN were included as candidate markers. Due to the nonsignificant subgroup differences in lesional cortex (reported in Supplementary Table 3), MD *Z*-scores of lesional cortex within networks are not included in the regression models as candidate markers here. Supplementary Table 4 shows the results of the linear regression model for integrity *Z*-scores (adjusted *R*^*2*^ = 0.22). Of the three included networks, the non-lesional cortex of the DMN was related to average cognition in people with MS in the final model [Β (95% confidence interval) = − 0.46 (− 0.86; − 0.43), *P* < 0.001; Fig. 4].

In order to evaluate the independence of integrity alterations in the non-lesional cortex concerning cognitive function in the context of CL volume, we introduced log(x + 1)-transformed CL volume of the DMN as an additional covariate in a distinct regression model aimed at elucidating average cognition. Results indicate that the mean *Z*-score MD of the non-lesional cortex in the default mode network [Β (95% confidence interval) = − 0.47 (− 0.70; − 0.24), *P* < 0.001] as well as cortical lesion volume [Β (95% confidence interval) = − 0.28 (− 0.44; − 0.13), *P* < 0.001] can independently explain variations in average cognition. Notably, the impact on cognition is more pronounced for non-lesional cortex MD compared to cortical lesion volume.

Additional regression analyses for functioning of individual cognitive domains showed that the non-lesional cortex of the DMN was significantly associated with information processing speed [Β (95% confidence interval) = − 0.96 (− 1.26; − 0.66), *P*_*corr*_ < 0.001], executive functioning—inhibition [Β (95% confidence interval) = − 0.63 (− 0.98; − 0.27), *P*_*corr*_ = 0.005], attention [Β (95% confidence interval) = − 0.55 (− 0.81; − 0.28), *P*_*corr*_ < 0.001], visuospatial memory [Β (95% confidence interval) = − 0.51 (− 0.76; − 0.26), *P*_*corr*_ < 0.001], and executive functioning—cognitive flexibility and verbal fluency [Β (95% confidence interval) = − 0.43 (− 0.70; − 0.15), *P*_*corr*_ = 0.01]; but not with working memory [Β (95% confidence interval) = − 0.30 (− 0.61; 0.02), *P*_*corr*_ = 0.43] and verbal memory [Β (95% confidence interval) = − 0.33 (− 0.58; − 0.09), *P*_*corr*_ = 0.06].

## Discussion

While CLs are known to strongly relate to cognitive impairment in MS, the relevance of normal-appearing cortex alterations remains unclear. This study investigated the pattern of structural (network) integrity loss in lesional and non-lesional cortex in people with MS, and related these integrity patterns to cognitive impairment. We showed lesional cortex in most people with MS, displaying increased FA and MD compared to non-lesional cortex. CL count and volume were higher in people with MS with worse cognition, particularly in the VAN. Cortical MD was increased in CI-MS compared to CP-MS, especially outside of lesions in the non-lesional cortex of the FPN, SMN and DMN. In contrast, cortical FA did not differ between cognitive phenotypes.

The cortex in people with MS with worse cognition was more affected by CLs, which is consistent with previous literature [9, 10, 24]. Lesional cortex had both an increased FA and MD compared to the non-lesional cortex, with a larger effect size of FA. FA increases might be induced by a disproportional loss of parallel axons in lesional cortex [6], followed by local tissue compaction. In comparison to healthy cortex, the FA values within the non-lesional cortex of our MS sample exhibited a trend towards a significant decrease. Previous research has reported lower FA in the normal-appearing cortex of people with MS compared to healthy controls, along with increased FA in cortical lesions compared to healthy controls [6]. These opposing changes in FA in the MS brain compared to healthy controls support the higher sensitivity to diffusivity alterations of our within-subject comparison between non-lesional and lesional cortex in people with MS. The lack of significance in non-lesional FA in our sample could be attributed to variations in the applied DIR protocol and the study population. As opposed to FA, increased MD values might indicate overall breakdown of microstructural barriers to diffusion, e.g., cell membranes [6, 22]. Hence, compared to MD, microstructural integrity reflected by FA might be more specific to focal cortical damage, which appears as a CL in the MS cortex. However, MD was more relevant in distinguishing cognitive phenotypes, and especially highlighted the relevance of non-lesional cortex compared to lesional cortex, which is supported by several previous studies [11, 12, 25]. These alterations of specifically MD might be reflective of, or even predate, volumetric changes in the form of atrophy [26], which is a major determinant of clinical and cognitive worsening in MS [27, 28]. The specific order of events and whether this hypothesized effect of microstructural integrity on volumetric changes is specific to non-lesional cortex compared to lesional cortex needs further investigation.

Looking at functional networks, the relative CL load was highest in the VAN, consisting of the insular and anterior cingulate cortices, frequently affected by cortical pathology in the MS brain [29]. CI-MS showed increased MD compared to CP-MS in SMN, FPN, DAN and DMN, with highest effect size in the DMN. Increases were particularly clear in non-lesional cortex. The effect on cognition seen in the sensorimotor cortex is potentially driven by more clinical disability in CI-MS compared to CP-MS [30], reflected in our cohort by higher disability scores in CI-MS. Previous fMRI studies highlighted the importance of alterations to the ‘task-active’ FPN and DAN and particularly the ‘task-negative’ DMN in disease progression and cognitive decline in MS [13, 14]. The VAN functions as a switch between the two [31]. Abnormal VAN connectivity seems to be relevant for cognitive impairment, potentially leading to aberrations in connected cognitive networks [13]. From a structural perspective, our findings might corroborate the concept that in MS, physiologic processes, reflected by microstructural integrity, relevant for maintaining overall network stability, are progressively disrupted as people with MS cognitively deteriorate [13]. In CI-MS, changes to specifically the DMN connectivity seems to predominate [13]. The DMN is thought to be stuck in a hyperconnected state, without being sufficiently inhibited by ‘task-active’ networks during cognitive tasks [32]. This could be a manifestation of the finding that the microstructural integrity in the DMN was most indicative for cognitive functioning in MS. Alterations in non-lesional diffusivity as well as cortical lesion volume of the DMN could independently explain cognitive functioning, indicating distinct contributions of both pathological processes to network disturbances. Follow-up studies are needed to demonstrate whether MD in the non-lesional cortex is directly related to functional connectivity in the MS brain along with the role of cortical lesions in this disruptive process.

This study has some limitations. The resolution of our DW images did not allow us to get more detailed spatial information regarding microstructural integrity in and surrounding CLs. Likewise, spatial characterization of GM diffusivity across various cortical layers would be an interesting future prospect if upcoming scanners would allow this microscopic resolution. Given the common resolution of diffusion protocol similar to ours, a more sophisticated approach to correct for partial volume effects, e.g., the use of advanced software to improve the image resolution, continues to be an important topic for future research. The detection of CLs has been substantially improved by the use of DIR sequences compared to clinical FLAIR sequences, but even using these advanced approaches the majority of CLs still go undetected, in particular subpial lesions [33]. We parcellated the brain into smaller regions in which we assessed the presence of CLs, rather than using the CL mask itself. This approach allowed us to control for physiologic diffusion heterogeneity relative to healthy controls and ensured that areas with CLs were considered as a single unit. Nevertheless, our areas with CLs did consist of both CLs and non-lesional cortex with a median of 13 out of 210 atlas regions showing CLs, which may have confounded our results. Also, future studies should investigate the effect of longitudinal white matter disconnection on non-lesional cortex integrity in functional networks, as white matter disconnection is found to affect structural and functional network functioning and cognition [34].

To conclude, most people with MS had CLs, while cognitive impairment was most strongly related to concurrent damage to non-lesional cortex. MD was more relevant to distinguish cognitive phenotypes compared to FA, while FA could best differentiate lesional from non-lesional tissue. CI-related damage to non-lesional cortex was most severe in the DMN, followed by the FPN and SMN, possibly indicating a preferential spatial susceptibility for cortical pathology relevant for cognitive decline.

### Supplementary Information

Below is the link to the electronic supplementary material.

Supplementary file1 (DOCX 106 KB)

## Data Availability

The tabulated data that support the findings of this study are available from the corresponding author, upon reasonable request from a qualified investigator.
