# Supplementary material for: The cognitive relevance of non-lesional damage to cortical networks in people with multiple sclerosis
Source: J Neurol. 2024 Mar 5;271(6):3203–14. doi: 10.1007/s00415-024-12240-4 (PMC11136718; doi:10.1007/s00415-024-12240-4)
Supplement: Supplementary file 1 — Supplementary file1 (DOCX 106 KB) [file 415_2024_12240_MOESM1_ESM.docx]

**Non-lesional damage to cortical networks is linked to cognitive impairment in people with multiple sclerosis**

Eva A. Krijnen, Tommy A.A. Broeders, Samantha Noteboom, Maureen van Dam, Albulena Bajrami, Piet M. Bouman, Frederik Barkhof, Bernard M.J. Uitdehaag, Eric. C. Klawiter, Ismail Koubiyr, Menno M. Schoonheim

**Corresponding author**: Eva A. Krijnen; MS Center Amsterdam, Anatomy and Neurosciences, Amsterdam Neuroscience, Amsterdam UMC location VUmc, 1007 MB Amsterdam, The Netherlands; Department of Neurology, Massachusetts General Hospital, Harvard Medical School, Boston, MA 02114, United States; E-mail: e.a.krijnen@amsterdamumc.nl

**Methods Supplement**

**Clinical assessments**

Neuropsychological data were obtained at the department of Neurology at Amsterdam UMC, supervised by M.M.S. and B.M.J.U. Neuropsychological data were classified into seven predefined cognitive domains: attention (reflected by card 1 and 2 of the Stroop color-word test[1], intercept of the memory comparison test[2] and concept shifting test parts A and B[3]), information processing speed (symbol digit modalities test[4]), working memory (slope of the memory comparison test[2]), visuospatial memory (the direct and delayed recall of the spatial recall test[5]), verbal memory [the long-term storage (first trial and total), consistent long term retrieval, and delayed recall of the selective reminding test[6]], executive functioning (EF) – cognitive flexibility and verbal fluency (the shifting score of the concept shifting test[3] and word list generation test[5]), and EF – inhibition (Stroop color-word test interference[1]). Raw test scores for each cognitive domain were corrected for age, sex and education based on normative data. Corrected test scores were transformed into Z-scores based on the Mean and standard deviation (SD) of the included healthy controls (HCs). People with multiple sclerosis (MS) were considered cognitively-impaired (CI) if they performed below -2.0 SD on two or more cognitive domains and mildly CI if they performed below -1.5 SD on two or more cognitive domains. Remaining people with MS not fulfilling any of these criteria were classified as cognitively-preserved (CP) [7, 8]. An average cognition Z-score was calculated based on the Z-scores of the seven cognitive domains.

**Data processing**

**White and grey matter segmentation**

Fluid attenuated inversion recovery images were used to segment white matter lesions in people with MS by means of k-Nearest-Neighbours approach with tissue type priors. White matter lesion maps were registered to 3D T1-weighted images and filled using LEAP for improving further 3D T1-weighted processing steps.[7] In order to segment our GM regions of interests (ROI), cortical surface reconstruction was carried out by FreeSurfer 7.0 software (https://surfer.nmr.mgh.harvard.edu/) with the use of the lesion-filled 3D T1-weighted images. All segmentations were checked, manually corrected and rerun if errors occurred. After surface reconstruction, the cortical grey matter (GM) was parcellated into 210 cortical GM regions (105 in each hemisphere) based on the Brainnetome atlas [9]. Partial volume fractions for the cortex and subcortical GM were computed using the *mri_compute_volume_fractions* function within FreeSurfer. The cortical Brainnetome atlas and partial volume maps were linearly transformed from FreeSurfer into participants’ native T1 space. In native T1 space, fourteen deep GM regions (seven in each hemisphere) were segmented using FSL FIRST, of which both hippocampi were added to the cortical atlas. This yielded a 3D T1 atlas for each participant consisting of 212 ROIs. All regions were grouped into seven networks based on the Yeo atlas to evaluate alterations in functionally related regions: sensorimotor, ventral attention, dorsal attention, frontoparietal, visual, limbic and default mode networks [10]. The allocation of regions to networks is shown in Supplementary Table 1.

**Cortical lesion segmentation**

According to the consensus guidelines developed by the MAGNIMS group[11], cortical lesions (CLs) were scored and segmented on double inversion recovery (DIR) images by P.M.B. (experienced neuroscientist), blinded to the patient characteristics, yielding a CL mask for each MS participant for lesion volumes and spatial distribution. Before scoring, DIR images were down-sampled to a 3mm slice thickness. DIR images were linearly registered to their corresponding 3D T1-weighted image with the use of FLIRT, after which the inverse matrix was applied to the 3D T1 atlas of each MS participant, using nearest-neighbour interpolation, to register the atlas to DIR space. By combining the CL mask and atlas, we were able to assess the presence and volume of CLs within each atlas ROI. Also, with the use of the *fslmeants* function in FSL, the ratio of the CL volume to the ROI volume in which the CL is located was calculated for all 212 cortical ROIs.

**Diffusion MRI Data**

DWI images were pre-processed by T.A.A.B. and M.M.S. with QSIPrep version 0.14.3, including head motion, eddy current and susceptibility distortion correction based on the ANTs symmetric normalization (SyN) technique [12]. Diffusion tensor fitting was applied to the pre-processed diffusion-weighted images for diffusion tensor imaging, yielding fractional anisotropy (FA) and mean diffusivity (MD) maps. ANTs transformation parameters were applied to the 3D T1 atlas and partial volume maps using nearest-neighbour and linear interpolation respectively to transform maps into diffusion space. To minimize the potential confounding by partial volume averaging effects in our analyses, we weighted mean FA and MD values within each atlas ROI by the partial volume fraction estimation.

Our regional network analyses of diffusion tensor imaging measurements of the cortex are complicated by the local cortical non-uniformity of diffusivity values, preventing straightforward interpretation of regional diffusivity and cytoarchitecture [13]. Therefore, we calculated regional FA and MD Z-scores per atlas ROI based on data of HCs for further network analyses to evaluate the potential integrity differences between subgroups and regions relative to physiologic diffusion heterogeneity.

**Statistical analysis**

**Within-subject integrity differences**

In people with MS, we sought to determine whether the microstructural integrity of lesional cortex differed from non-lesional cortex and if so, whether this integrity alteration varied among cognitive subgroups. Within-subject mean global FA and MD alterations between ROIs with CL and ROIs with non-lesional cortex were assessed by paired t-tests in the overall MS cohort. If significant, paired t-tests were performed within cognitive subgroups.

**Between-group global differences**

The CL distribution was assessed within functionally-related regions and among cognitive subgroups by means of univariate linear models and multinomial logistic regression models, adjusting for age, sex and level of education.

We compared mean FA and MD in non-lesional cortex between people with MS and HCs. Mean global FA and MD were further analysed between cognitive groups (CP, mildly CI and CI) using the same approach, including post-hoc tests to assess the significance of differences between pairs of group means. To test whether these integrity alterations between cognitive subgroups are driven by ROIs with CL and/or ROIs consisting of solely non-lesional cortex, significant between-group differences were further explored by including both the integrity measures in ROIs with CL and ROIs with non-lesional cortex as dependent variables in the linear models. Independent variables in these multivariate linear models included the cognitive subgroups and the confounders age, sex and level of education. In this approach, only people with MS with CLs were included, eliminating potential bias from people with MS without CLs.

**Between-group spatial integrity differences within functional networks**

Significant between-group differences in cortical integrity were also spatially analysed among the seven functional networks. In order to limit bias from healthy spatial variance in microstructure, only the Z-scores of integrity measures were used for this part of the statistical analyses. The mean integrity Z-scores that show significant differences at whole-brain level were compared between cognitive subgroups within each of the seven networks. In these follow-up analyses within networks, only people with MS that showed CLs in that specific network were included in the analysis. In networks showing significant integrity differences between cognitive groups, Z-score differences were further assessed by including both the mean integrity of ROIs with CL and of ROIs with non-lesional cortex as dependent variables to evaluate which of the two regions contributes most to the significant integrity difference at network-level.

To assess which significant networks best explain the variability in cognitive functioning in people with MS, the integrity Z-scores of those networks were included in a multivariable linear regression model, explaining average cognition Z-score. Demographics (age, sex, and level of education) were entered in a single step, and the candidate integrity Z-scores were included in a backward stepwise selection block, applying a probability of F thresholds of 0.05 (entry) and 0.10 (removal) as stepping method criteria. The network measure best explaining average cognition was additionally related to individual cognitive domains.

**References**

[1] Van der Elst W, Van Boxtel MP, Van Breukelen GJ, Jolles J (2008) Detecting the significance of changes in performance on the Stroop Color-Word Test, Rey's Verbal Learning Test, and the Letter Digit Substitution Test: the regression-based change approach. J Int Neuropsychol Soc 14:71-80. https://doi.org/10.1017/S1355617708080028.

[2] Van Der Elst W, Van Boxtel MP, Van Breukelen GJ, Jolles J (2007) Assessment of information processing in working memory in applied settings: the paper and pencil memory scanning test. Psychol Med 37:1335-1344. https://doi.org/10.1017/S0033291707000360.

[3] Van der Elst W, Van Boxtel MP, Van Breukelen GJ, Jolles J (2006) The Concept Shifting Test: adult normative data. Psychol Assess 18:424-432. https://doi.org/10.1037/1040-3590.18.4.424.

[4] Smith A (1982) Symbol Digits Modalities Test. In:Western Psychological Services,  Los Angeles.

[5] Rao S (1990) A manual for brief repeatable battery of the neuropsychological tests in multiple sclerosis. In:Milwaukee:  Medical College of Wisconsin.

[6] Buschke H, Fuld PA (1974) Evaluating storage, retention, and retrieval in disordered memory and learning. Neurology 24:1019-1025. https://doi.org/10.1212/wnl.24.11.1019.

[7] Eijlers AJC, Wink AM, Meijer KA, Douw L, Geurts JJG, Schoonheim MM (2019) Reduced Network Dynamics on Functional MRI Signals Cognitive Impairment in Multiple Sclerosis. Radiology 292:449-457. https://doi.org/10.1148/radiol.2019182623.

[8] Schoonheim MM, Hulst HE, Brandt RB, Strik M, Wink AM, Uitdehaag BM, Barkhof F, Geurts JJ (2015) Thalamus structure and function determine severity of cognitive impairment in multiple sclerosis. Neurology 84:776-783. https://doi.org/10.1212/WNL.0000000000001285.

[9] Fan L, Li H, Zhuo J, Zhang Y, Wang J, Chen L, Yang Z, Chu C, Xie S, Laird AR, Fox PT, Eickhoff SB, Yu C, Jiang T (2016) The Human Brainnetome Atlas: A New Brain Atlas Based on Connectional Architecture. Cereb Cortex 26:3508-3526. https://doi.org/10.1093/cercor/bhw157.

[10] Yeo BT, Krienen FM, Sepulcre J, Sabuncu MR, Lashkari D, Hollinshead M, Roffman JL, Smoller JW, Zöllei L, Polimeni JR, Fischl B, Liu H, Buckner RL (2011) The organization of the human cerebral cortex estimated by intrinsic functional connectivity. J Neurophysiol 106:1125-1165. https://doi.org/10.1152/jn.00338.2011.

[11] Geurts JJ, Roosendaal SD, Calabrese M, Ciccarelli O, Agosta F, Chard DT, Gass A, Huerga E, Moraal B, Pareto D, Rocca MA, Wattjes MP, Yousry TA, Uitdehaag BM, Barkhof F, Group MS (2011) Consensus recommendations for MS cortical lesion scoring using double inversion recovery MRI. Neurology 76:418-424. https://doi.org/10.1212/WNL.0b013e31820a0cc4.

[12] Treiber JM, White NS, Steed TC, Bartsch H, Holland D, Farid N, McDonald CR, Carter BS, Dale AM, Chen CC (2016) Characterization and Correction of Geometric Distortions in 814 Diffusion Weighted Images. PLoS One 11:e0152472. https://doi.org/10.1371/journal.pone.0152472.

[13] Kang X, Herron TJ, Turken AU, Woods DL (2012) Diffusion properties of cortical and pericortical tissue: regional variations, reliability and methodological issues. Magn Reson Imaging 30:1111-1122. https://doi.org/10.1016/j.mri.2012.04.004.

**Supplementary Table 1**

| Functional network | Brainnetome atlas regions |
| --- | --- |
| Sensori-  motor | Left and right medial area 6; Left and right area 4 (head and face region); Left and right area 4 (upper limb region); Left and right area 4 (trunk region); Left and right area1/2/3 (lower limb region); Left and right area 4 (lower limb region); Left and right area 41/42; Left and right TE1.0 and TE1.2; Left and right caudal area 22; Right rostral area 22; Left and right postcentral area 7; Left and right area 1/2/3 (upper limb, head and face region); Left and right area 1/2/3; Left and right area 2; Left and right area1/2/3 (trunk region); Left and right hypergranular insula; Left and right dorsal granular insula |
| Ventral attention | Left and right medial area 8; Left dorsal area 9/46; Left and right opercular area 44; Left and right area 4 (tongue and larynx region); Right caudoposterior superior temporal sulcus; Left and right rostroventral area 40 (PFop); Left medial area 5 (PEm); Right ventral agranular insula; Left and right dorsal agranular insula; Left and right ventral dysgranular and granular insula; Left and right dorsal dysgranular insula; Right pregenual area 32; Left and right caudodorsal area 24; Left and right caudal area 23 |
| Dorsal attention | Left and right dorsolateral area 6; Left ventrolateral area 6; Left and right caudal dorsolateral area 6; Left and right caudal ventrolateral area 6; Left and right dorsolateral area37; Left and right extreme lateroventral area37; Left and right ventrolateral area 37; Left lateroventral area37; Left and right rostral area 7; Left and right caudal area 7; Left and right lateral area 5; Left and right intraparietal area 7 (hIP3); Right caudal area 39 (PGp); Left and right rostrodorsal area 40 (PFt); Right medial area 7 (PEp); Right medial area 5 (PEm) |
| Fronto-parietal | Right dorsal area 9/46; Left and right inferior frontal junction; Left and right area 46; Left and right ventral area 9/46; Right ventrolateral area 8; Right ventrolateral area 6; Right lateral area10; Left and right dorsal area 44; Left and right inferior frontal sulcus; Right caudal area 45; Right rostral area 45; Right ventral area 44; Left and right caudolateral of area 20; Left and right rostrodorsal area 39 (Hip3); Right caudal area 40 (PFm); Left medial area 7 (PEp); Left rostroventral area 24 |
| Visual | Left and right medioventral area37; Right lateroventral area37; Right area TL (lateral posterior parahippocampal gyrus); Left and right area TH (medial PPHC); Left caudal area 39 (PGp); Right dorsomedial parietooccipital sulcus (PEr); Left and right caudal lingual gyrus; Left and right rostral cuneus gyrus; Left and right caudal cuneus gyrus; Left and right rostral lingual gyrus; Left and right ventromedial parietooccipital sulcus; Left and right middle occipital gyrus; Left and right area V5/MT+; Left and right occipital polar cortex; Left and right inferior occipital gyrus; Left and right medial superior occipital gyrus; Left and right lateral superior occipital gyrus |
| Limbic | Left and right lateral area 11; Left and right medial area 11; Left and right area 13; Left and right medial area 38; Left and right lateral area 38; Left and right intermediate ventral area 20; Left and right rostral area 20; Right intermediate lateral area 20; Left and right caudoventral of area 20; Left and right rostroventral area 20; Left and right rostral area 35/36; Left and right caudal area 35/36; Left and right area 28/34 (entorhinal cortex); Left and right area TI (temporal agranular insular cortex) |
| Default  mode | Left and right dorsolateral area 8; Left and right lateral area 9; Left and right medial area 9; Left and right medial area 10; Left ventrolateral area 8; Left lateral area10; Left caudal area 45; Left rostral area 45; Left ventral area 44; Left and right medial area 14; Left and right orbital area 12/47; Left and right lateral area 12/47; Left rostral area 22; Left and right caudal area 21; Left and right rostral area 21; Left and right anterior superior temporal sulcus; Left intermediate lateral area 20; Left area TL (lateral posterior parahippocampal gyrus); Left and right rostroposterior superior temporal sulcus; Left caudoposterior superior temporal sulcus; Left caudal area 40 (PFm); Left and right rostroventral area 39 (PGa); Left dorsomedial parietooccipital sulcus (PEr); Left and right area 31 (Lc1); Left ventral agranular insula; Left and right dorsal area 23; Right rostroventral area 24; Left pregenual area 32; Left and right ventral area 23; Left and right subgenual area 32; left and right hippocampus |

Allocation of 210 Brainnetome atlas^1^ regions, including the hippocampi of FSL FIRST segmentation, into seven functional networks based on the Yeo atlas^2^.

^1^Fan L, Li H, Zhuo J, et al. The Human Brainnetome Atlas: A New Brain Atlas Based on Connectional Architecture. *Cereb Cortex*. 08 2016;26(8):3508-26. doi:10.1093/cercor/bhw157

^2^Yeo BT, Krienen FM, Sepulcre J, et al. The organization of the human cerebral cortex estimated by intrinsic functional connectivity. *J Neurophysiol*. Sep 2011;106(3):1125-65. doi:10.1152/jn.00338.2011

**Supplementary Table 2**

| **Integrity measure** | **CP-MS**  **N=80** | | *NA vs. lesional cortex^a^* | **Mildly CI-MS**  **N=35** | | *NA vs. lesional cortex^a^* | **CI-MS**  **N=39** | | *NA vs. lesional cortex^a^* |
| --- | --- | --- | --- | --- | --- | --- | --- | --- | --- |
|  | NA  cortex | Lesional cortex |  | NA  cortex | Lesional cortex |  | NA  cortex | Lesional cortex |  |
| **Mean diffusivity** | 1.05  (0.04) | 1.07  (0.08) | *t(79)=1.89,*  *p=.06* | 1.07  (0.04) | 1.08  (0.06) | *t(34)=0.88,*  *p=.38* | 1.09  (0.05) | 1.11  (0.09) | *t(38)=2.40,*  *p=.02* |
| **Fractional anisotropy** | 0.17  (0.01) | 0.18  (0.02) | *t(79)=3.10,*  *p=.003** | 0.17  (0.01) | 0.18  (0.01) | *t(34)=2.04,*  *p=.05* | 0.17  (0.01) | 0.18  (0.01) | *t(38)=2.79, p=.008** |

Raw integrity values in normal-appearing (NA) and lesional cortex of people with multiple sclerosis (MS) per cognitive subgroup: cognitively-preserved (CP), mildly cognitively-impaired (CI) and CI patients with multiple sclerosis (MS). Variables are reported as Mean (SD). Raw unadjusted p-values are shown.

^a^Paired t-test between non-lesional and lesional cortex in people with MS with cortical lesions per cognitive subgroup. T-test statistics with corresponding p-values are reported.

**P*-value surviving Bonferroni correction (*p*<8.33⋅10^-3^).

**Supplementary Table 3**

| **Functional network** | **CP-MS** | **CI-MS** | ***CP vs. CI^a,b^*** |
| --- | --- | --- | --- |
| Ventral attention network |  |  |  |
| *Overall* | 0.54 (0.58) | 0.84 (0.79) | *F(1,91)=6.50, p=.01* |
| *NA cortex* | 0.52 (0.57) | 0.83 (0.80) | *F(1,91)=6.34, p=.01* |
| *Lesional cortex* | 0.49 (0.90) | 0.91 (1.00) | *F(1,91)=6.12, p=.02* |
| Dorsal attention network |  |  |  |
| *Overall* | 0.41 (0.54) | 0.83 (0.79) | *F(1,76)=8.89, p=.004** |
| *NA cortex* | 0.42 (0.60) | 0.81 (0.83) | *F(1,76)=6.85, p=.01* |
| *Lesional cortex* | 0.50 (0.92) | 0.85 (1.03) | *F(1,76)=2.35, p=.13* |
| Sensorimotor network |  |  |  |
| *Overall* | 0.32 (0.56) | 0.68 (0.68) | *F(1,85)=8.02, p=.006** |
| *NA cortex* | 0.31 (0.55) | 0.69 (0.77) | *F(1,85)=7.66, p=.007** |
| *Lesional cortex* | 0.43 (0.92) | 0.75 (0.69) | *F(1,85)=2.89, p=.09* |
| Visual network |  |  |  |
| *Overall* | 0.79 (0.75) | 0.99 (0.69) | *F(1,42)=0.53, p=.47* |
| *NA cortex* | 0.81 (0.81) | 0.95 (0.71) |  |
| *Lesional cortex* | 0.82 (1.23) | 1.34 (1.07) |  |
| Limbic network |  |  |  |
| *Overall* | 0.43 (0.46) | 0.69 (0.56) | *F(1,83)=4.54, p=.04* |
| *NA cortex* | 0.46 (0.48) | 0.63 (0.54) | *F(1,83)=1.67, p=.03* |
| *Lesional cortex* | 0.32 (0.62) | 0.68 (1.02) | *F(1,83)=4.74, p=.20* |
| Default mode network |  |  |  |
| *Overall* | 0.54 (0.56) | 1.03 (0.73) | *F(1,97)=14.12, p<.001** |
| *NA cortex* | - 1. 0.50) | 1.01 (0.76) | *F(1,97)=15.55, p<.001** |
| *Lesional cortex* | 0.67 (0.94) | 1.08 (0.97) | *F(1,97)=6.64, p=.01* |
| Frontoparietal network |  |  |  |
| *Overall* | 0.37 (0.56) | 0.84 (0.73) | *F(1,73)=8.74, p=.004** |
| *NA cortex* | 0.37 (0.55) | 0.81 (0.66) | *F(1,73)=8.46, p=.005** |
| *Lesional cortex* | 0.26 (0.78) | 0.92 (1.35) | *F(1,73)=7.24, p=.009* |

Mean diffusivity Z-scores in normal-appearing (NA) and lesional cortex relative to healthy volunteers in cognitively-preserved (CP) and cognitively-impaired (CI) patients with multiple sclerosis (MS). Variables are reported as Mean (SD). Raw unadjusted p-values are shown.

^a^Univariate linear model for whole-brain integrity, adjusting for age, sex and high level of education. Only people with MS with cortical lesions in a specific network were included. F-test statistics with corresponding p-values are reported.

^b^Multivariate linear model for whole-brain integrity in both non-lesional and lesional cortex, adjusting for age, sex and high level of education. Using this model, only people with MS with CLs were included, eliminating potential bias from people with MS without CLs. F-test statistics with corresponding p-values are reported.

**P*-value surviving Bonferroni correction (*p*<7.14⋅10^-3^ in “overall” network analyses; *p*<8.33⋅10^-3^ in non-lesional and lesional cortex network analyses)

**Supplementary Table 4**

| **Variables** | **Linear regression model for average cognition^a^** |
| --- | --- |
| Age | 0.02 (-0.01 – 0.02), p=.81 |
| Sex | -0.07 (-0.44 – 0.16), p=.35 |
| High level of education | 0.17 (0.03 – 0.61), p=.03 |
| Default mode network NA cortex | -0.46 (-0.86 – -0.43), p<.001 |

^a^Multivariable linear regression model explaining average cognition with integrity Z-scores of normal-appearing (NA) cortex in cortical networks as independent variables, adjusting for age, sex and high level of education. Demographics were entered in a single step; candidate integrity Z-scores were included in a backward stepwise selection block. Standardized Beta-coefficients (β) with 95% confidence intervals of variables included in the final model are reported with corresponding *p*-value.

**Supplementary Figure 1**

**
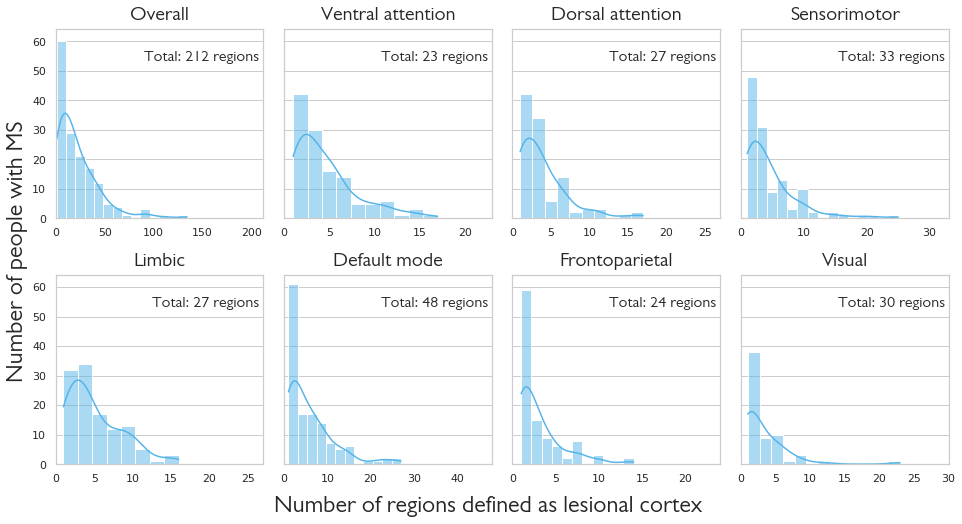
**

Histograms including kernel density estimates (blue lines) of the distribution of number of regions defined as lesional cortex in the entire cortex, referred to as ‘overall’, as well as in all functional networks separately in people with multiple sclerosis (MS): ventral attention, dorsal attention, sensorimotor, limbic, default mode, frontoparietal, and visual network. People with MS without cortical lesions were excluded from the plots.
